# Longitudinal extensive transverse myelitis following ChAdOx1 nCOV-19 vaccine: a case report

**DOI:** 10.1186/s12883-021-02427-x

**Published:** 2021-10-12

**Authors:** Wee Yong Tan, Abdul Hanif Khan Yusof Khan, Mohd Naim Mohd Yaakob, Anna Misyail Abdul Rashid, Wei Chao Loh, Janudin Baharin, Azliza Ibrahim, Mohd Redzuan Ismail, Liyana Najwa Inche Mat, Wan Aliaa Wan Sulaiman, Hamidon Basri, Fan Kee Hoo

**Affiliations:** 1 Thomson Hospital Kota Damansara, 47810 Kuala Lumpur, Malaysia; 2 Department of Neurology, Faculty of Medicine and Health Sciences, Universiti Putra Malaysia (UPM), 43400 Serdang, Selangor Malaysia; 3 Department of Radiology, Faculty of Medicine and Health Sciences, Universiti Putra Malaysia (UPM), 43400 Serdang, Selangor Malaysia

**Keywords:** Myelitis, transverse, COVID-19, Vaccination, Malaysia, Case report

## Abstract

**Background:**

Transverse myelitis (TM) is a relatively uncommon condition, and vaccine-associated myelitis is even rarer. Concern regarding neurological complications following vaccination escalated following the report of TM during the safety and efficacy trials of the COVID-19 vaccine.

**Case presentation:**

We report the first case of Longitudinal Extensive Transverse Myelitis (LETM) in Malaysia following administration of the chimpanzee adenovirus-vectored (ChAdOx1 nCoV-19) vaccine. A 25-year-old female presented with bilateral lower limb weakness and inability to walk with a sensory level up to T8 with absent visual symptoms. Urgent gadolinium-enhanced magnetic resonance imaging (MRI) of the spine showed long segment TM over the thoracic region. Cerebrospinal fluid autoantibodies for anti-aquaporin-4 and anti-myelin-oligodendrocyte were negative. A diagnosis of LETM following vaccination was made, and the patient was started on a high dose of intravenous methylprednisolone. The patient eventually made a recovery following treatment.

**Conclusion:**

LETM is a rare but serious adverse reaction following vaccination. Previously reported cases showed an onset of symptoms between 10 to 14 days post-vaccination, suggesting a delayed immunogenic reaction. However, the incidence of myelitis in COVID-19 is much more common, far greater than the risk associated with vaccination.

## Background

In the biggest vaccination campaign of our times, more than 3 billion doses of COVID-19 vaccines have been administered worldwide thus far [1]. Concern regarding neurological complications reported as an adverse event following immunisation (AEFI) escalated following the report of two participants during the chimpanzee adenovirus-vectored (ChAdOx1 nCoV-19) vaccine (AZD1222) safety and efficacy trial developing transverse myelitis (TM) [2, 3]. Although it was deemed unlikely to be related to vaccination (pre-existing multiple sclerosis) in the first patient, the development of idiopathic, short segment spinal cord demyelination in the second patient, which occurred 14 days following booster vaccination, was possibly vaccine-related. We report the first case of LETM in Malaysia following the administration of the recombinant, viral-vectored ChAdOx1 nCOV-19 vaccine.

## Case presentation

The patient is a 25-year-old female with no comorbidities who presented to the emergency department on day 16 following her first dose of the ChAdOx1 nCOV-19 vaccine complaining of inability to walk and urinary retention. She developed fever during the first 48 h following the vaccination, which subsequently resolved. From day five onwards, she complained of myalgia of her lower limbs, especially upon walking up stairs. There was a further onset of fever with progressive bilateral lower limb weakness on day 12. By day 16, she was unable to stand and developed urinary retention. There were no visual, respiratory or gastrointestinal symptoms. On examination, she was afebrile with normal vital signs and had numbness and allodynia below the T8 spinal level. In the lower limbs, there was bilateral hypertonia with reduced power (3/5 proximally and distally) along with exaggerated deep tendon reflexes at the knees and ankles with upgoing plantar. Upper limb and cranial nerve examinations were normal, along with no evidence of optic neuritis or cerebellar signs. Blood investigations revealed haemoglobin of 15.0 g/dL with total white cells of 8.12 x 10^3^μL (81% neutrophils and 15% lymphocytes) and platelets of 285 x 10^3^μL. Renal and liver functions test were normal. Erythrocytes sedimentation rate (ESR) was 21 mm/hr. (upper limit normal). Urine microscopy revealed the presence of leucocytes and bacteria, which culture eventually showed no growth.

**Fig. 1:**
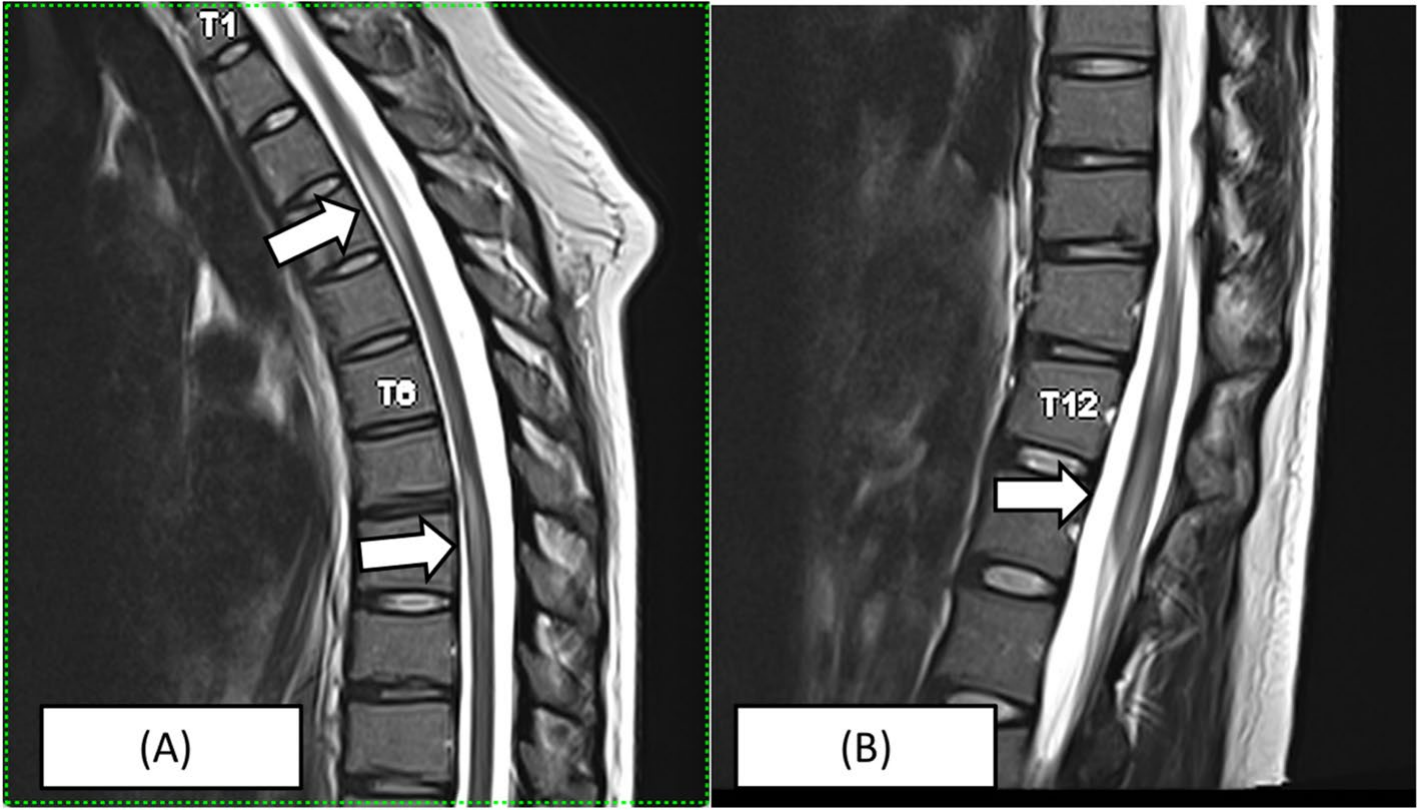
MRI spine of the patient. **A** T2 sagittal view showing expansile hyperintense lesion at the T3 – T5 level and T7 – T9 level. **B** T2 sagittal view showing slight central hyperintense lesion at T12 – L1 level

**Fig. 2:**
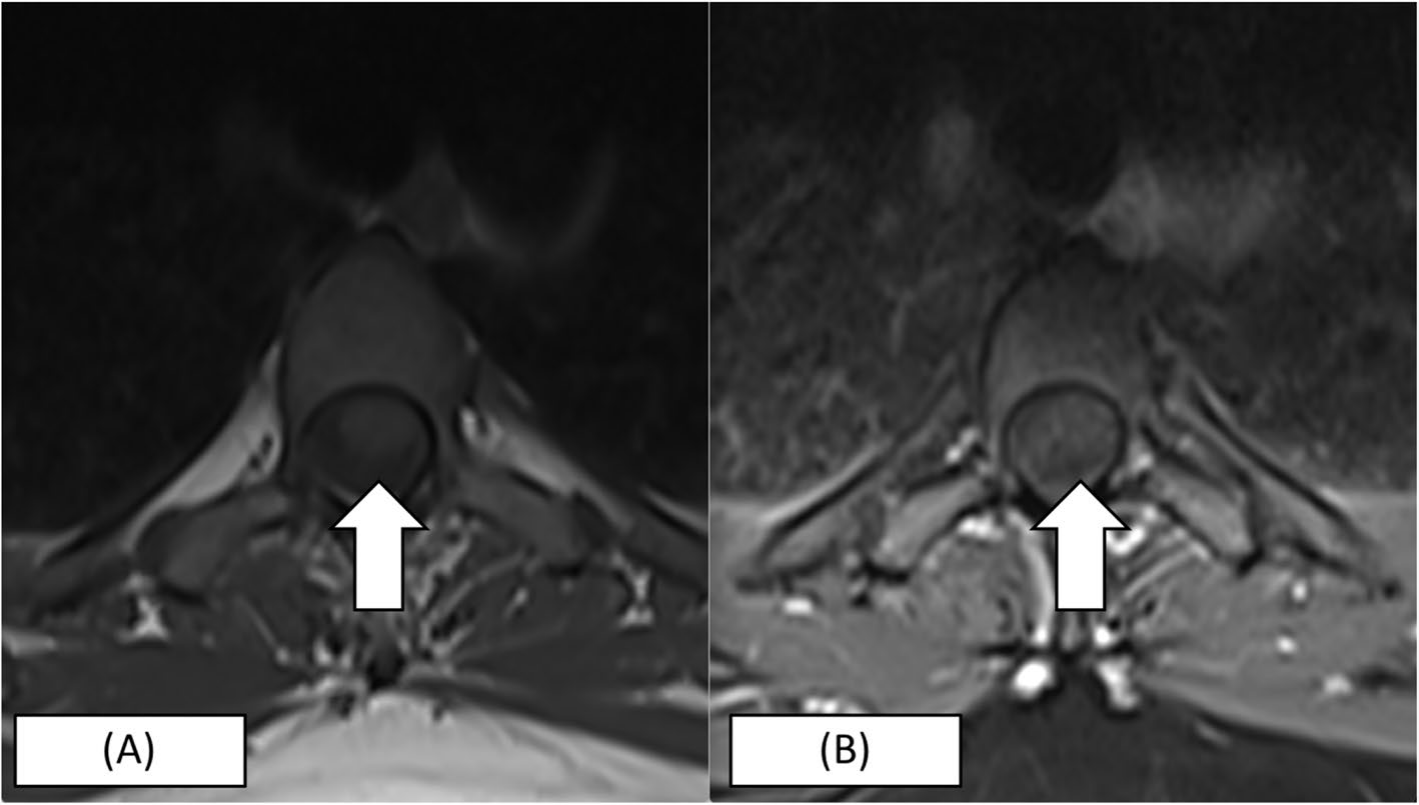
**A** T1 Axial Plain at T4 level which shows (**B**) variable cord enhancement of the lesion (more than 2/3 of the cord) in post contrast images

**Fig. 3:**
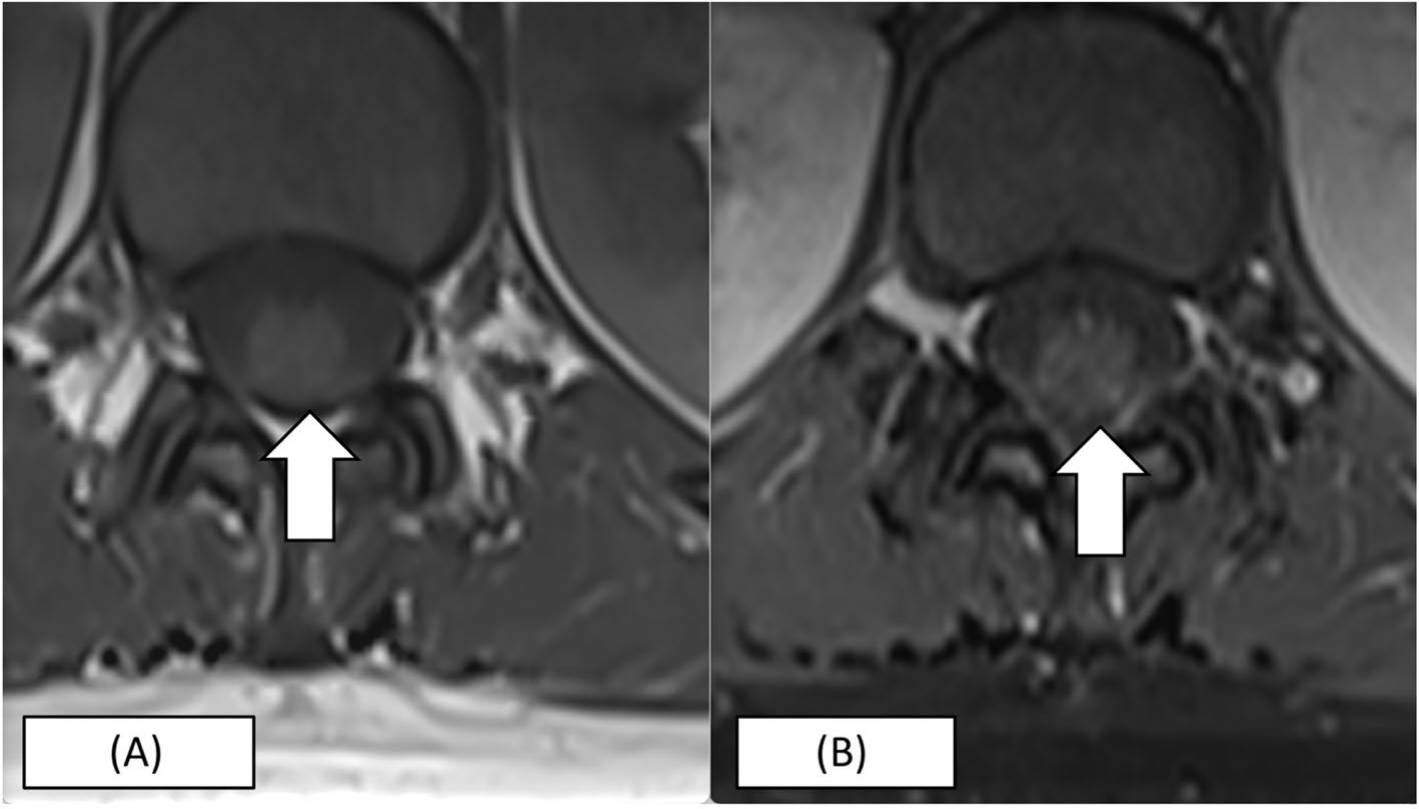
**A** T1 Axial Plain at T12 level which shows (**B**) variable cord enhancement of the lesion in post contrast images (more than 2/3 of the cord)

Urgent gadolinium-enhanced magnetic resonance imaging (MRI) of the whole spine revealed multi-segment T2-hyperintensities (T3-T5, T7-T8 and T11-L1), which showed variable cord enhancement post-contrast at T7-T8 lesions (Figs. 1, 2, and 3). Visualised MRI brain was normal. Cerebrospinal fluid (CSF) examination showed clear-appearing CSF with an elevated protein count of 546 mg/L (normal range: 150 -400) and CSF glucose of 3.1 mmol/L (serum glucose of 5.6 mmol/L). No acid-fast bacilli were seen, and no growth was obtained from CSF and blood culture. CSF anti-aquaporin 4 (AQ-4) and anti-myelin-oligodendrocyte were negative. No oligoclonal bands were detected in CSF. Connective tissue screening, including antinuclear antibody and rheumatoid factor, were negative. Somatosensory evoked potential (SSEP) and nerve conduction study were normal. She was diagnosed with LETM following COVID-19 vaccination and started on intravenous methylprednisolone 1000 mg daily for 5 days. The patient was also started on intravenous ceftriaxone covering for urinary tract infection for 5 days and subcutaneous enoxaparin for deep venous thrombosis prophylaxis. The patient responded to the treatment and was discharged after 1 week. Upon discharge, the patient was able to walk about ten steps but was still limited given ongoing pain and numbness, mainly at the distal lower limb up to the knee. Her urinary incontinence resolved. Two weeks later, the patient was able to walk without assistance; however, she still had persistent neuropathic pain up to the knees bilaterally. She was understandably distraught, but at the same time, encouraged by her gradually improving condition. She will continue to be on a regular follow-up to observe the recurrence of symptoms and monitor recovery. Despite the adverse event, the patient is keen on the COVID-19 vaccine, where we suggested an mRNA based vaccine in 1 month following the index event.

## Discussion

TM is a relatively uncommon condition with an estimated incidence between 1.34 and 4.6 cases per million annually [4], and vaccine-associated myelitis is even rarer. Nine cases of TM were reported in the Centers for Disease Control (CDC)‘s Vaccine Adverse Event Reporting System (VAERS) related to Pfizer-BioNTech, Moderna and Johnson & Johnson’s COVID-19 vaccine [5]. One case has been deemed likely related to the ChAdOx1 nCOV-19 (AstraZeneca™) vaccine during the efficacy and safety trials [2, 3]. Along with previously reported cases, our case suggests a temporal relationship of symptom-onset between 10 to 14 days following ChAdOX1 nCOV-19 vaccine administration, possibly due to delayed immunological reactions [2, 3, 6–8]. In addition, possible molecular mimicry by the viral vector may induce autoimmunity by a cascade of inflammatory reactions propagated via dendritic and T-cells [7]. In other vaccines, such as alumuinium-containing vaccines, adjuvants used to amplify the immune response have been associated with autoimmunity [7]. However, the ChAdOX1 nCOV-19 vaccine does not contain adjuvant or preservatives.

LETM is diagnosed when there are contiguous central cord lesions with variable contrast enhancement extending over three or more vertebral segments on spinal MRI [9]. Patients typically have a dramatic presentation of acute or subacute paraparesis or tetraparesis, with sensory disturbances and alteration of gait, bladder, bowel and sexual dysfunction, depending on the location of lesion on the spinal cord [9]. Although our case suggests temporal causality between the ChAdOX1 nCOV-19 vaccine and LETM, other differentials had to be ruled out in particular neuromyelitis optica spectrum disorder (NMOSD), multiple sclerosis (MS), and infectious causes [9]. The absence of both autoantibodies (AQ-4 and MOG) combined with the absence of optic neuritis make the diagnosis of NMOSD unlikely. Furthermore, the absence of CSF pleocytosis, typical lesion of MS in the spine (usually patchy involving one or two vertebrae with peripheral enhancement), negative oligoclonal bands and normal MRI brain are not consistent with MS. However, this patient need to be followed up for the reoccurrence of symptoms in the future as this could be the first isolated lesion (clinically isolated syndrome) in both relapsing-remitting courses of MS. Lastly, sterile CSF with relatively normal inflammatory markers were not suggestive of infectious myelitis. Based on the currently available evidence and the temporal relationship, the occurrence of LETM follwing vaccination, in this case, was probable based on the adverse drug reaction probability scale (point + 6) [10].

A recent review showed that the prevalence of myelitis in COVID-19 patients is far greater, accounting for approximately 1.2% of all COVID-19 neurological related complications where LETM accounted for 70% of the reported TM in the series [11]. Although unfortunate, patients who have developed LETM following vaccines have responded relatively well to treatment, suggesting what is hopefully a temporary immunogenic reaction.

## Conclusion

Although TM following vaccination is rare, the temporal causality of LETM, in this case, is undeniable. However, this should not deter us from continuing to recommend COVID-19 vaccines, as the incidence of myelitis in COVID-19 is much higher.

## Data Availability

The data and images used in this case report are available from the corresponding author on reasonable request.

## Abbreviations

AEFI: Adverse Event Following Immunisation
AQ-4: Anti-aquaporin 4
ChAdOx1 nCoV-19 vaccine: Chimpanzee adenovirus-vectored vaccine
CDC: Centers for Disease Control
CSF: Cerebrospinal Fluid
TM: Transverse Myelitis
LETM: Longitudinal Extensive Transverse Myelitis
MRI: Magnetic Resonance Imaging
MOG: Anti-myelin-oligodendrocyte
MS: Multiple sclerosis
NMOSD: Neuromyelitis Optica Spectrum Disorder
SSEP: Somatosensory Evoked Potential
VAERS: Vaccine Adverse Event Reporting System
